# Phasic, Event-Related Transcutaneous Auricular Vagus Nerve Stimulation Modifies Behavioral, Pupillary, and Low-Frequency Oscillatory Power Responses

**DOI:** 10.1523/JNEUROSCI.0452-23.2023

**Published:** 2023-09-06

**Authors:** Christian Wienke, Marcus Grueschow, Aiden Haghikia, Tino Zaehle

**Affiliations:** ^1^Otto-von-Guericke University, 39120 Magdeburg, Germany; ^2^Zurich Center for Neuroeconomics, Departement of Economics, University of Zurich, 8006 Zurich, Switzerland; ^3^Deusches Zentrum für Neurodegenrative Erkrankungen, 39120 Magdeburg, Germany; ^4^Center for Behavioral Brain Sciences, Magdeburg, 39120, Germany

**Keywords:** EEG/MEG, noradrenalin, pupil, pupil light reflex, taVNS, transcutaneous vagus nerve stimulation

## Abstract

Transcutaneous auricular vagus nerve stimulation (taVNS) has been proposed to activate the locus ceruleus-noradrenaline (LC-NA) system. However, previous studies failed to find consistent modulatory effects of taVNS on LC-NA biomarkers. Previous studies suggest that phasic taVNS may be capable of modulating LC-NA biomarkers such as pupil dilation and alpha oscillations. However, it is unclear whether these effects extend beyond pure sensory vagal nerve responses. Critically, the potential of the pupillary light reflex as an additional taVNS biomarker has not been explored so far. Here, we applied phasic active and sham taVNS in 29 subjects (16 female, 13 male) while they performed an emotional Stroop task (EST) and a passive pupil light reflex task (PLRT). We recorded pupil size and brain activity dynamics using a combined Magnetoencephalography (MEG) and pupillometry design. Our results show that phasic taVNS significantly increased pupil dilation and performance during the EST. During the PLRT, active taVNS reduced and delayed pupil constriction. In the MEG, taVNS increased frontal-midline theta and alpha power during the EST, whereas occipital alpha power was reduced during both the EST and PLRT. Our findings provide evidence that phasic taVNS systematically modulates behavioral, pupillary, and electrophysiological parameters of LC-NA activity during cognitive processing. Moreover, we demonstrate for the first time that the pupillary light reflex can be used as a simple and effective proxy of taVNS efficacy. These findings have important implications for the development of noninvasive neuromodulation interventions for various cognitive and clinical applications.

**SIGNIFICANCE STATEMENT** taVNS has gained increasing attention as a noninvasive neuromodulation technique and is widely used in clinical and nonclinical research. Nevertheless, the exact mechanism of action of taVNS is not yet fully understood. By assessing physiology and behavior in a response conflict task in healthy humans, we demonstrate the first successful application of a phasic, noninvasive vagus nerve stimulation to improve cognitive control and to systematically modulate pupillary and electrophysiological markers of the noradrenergic system. Understanding the mechanisms of action of taVNS could optimize future clinical applications and lead to better treatments for mental disorders associated with noradrenergic dysfunction. In addition, we present a new taVNS-sensitive pupillary measure representing an easy-to-use biomarker for future taVNS studies.

## Introduction

Transcutaneous auricular vagus nerve stimulation (taVNS) has gained increasing attention as a noninvasive neuromodulation technique in recent years. Since the seminal work by Peuker and Filler (2002) describing that areas of the human outer ear are exclusively innervated by the auricular branch of the vagus nerve, taVNS has been widely used in clinical and nonclinical research settings (for review, see Burger et al., 2020b; Farmer et al., 2021), but its exact working mechanisms are still not fully understood.

It has been suggested that taVNS modulates the locus ceruleus-noradrenaline (LC-NA) system, which is involved in various cognitive and emotional processes (Kurniawan et al., 2021; Maier and Grueschow, 2021). The LC-NA system receives indirect input from the VN through projections from the brainstem nucleus tractus solitarius (NTS; Butt et al., 2020). The LC is the main source of NA in the brain (Sara, 2009) and invasive VNS (iVNS) in animals modulated LC firing and cortical NA levels (Raedt et al., 2011; Hulsey et al., 2017).

Pupil dilation (PD) has been identified as a promising indicator of noradrenergic activity given its close relation to the LC-NA system (Samuels and Szabadi, 2008a, 2008b; Joshi et al., 2016). In humans, the LC signals behavioral response conflicts, accompanied by an increase in PD (Grueschow et al., 2021). Direct LC stimulation (Joshi et al., 2016) and iVNS in animals (Mridha et al., 2021) and humans (Desbeaumes Jodoin et al., 2015) also increased PD. It is important to note, however, that NA is not the only transmitter involved in regulating PD (Reimer et al., 2016; Mridha et al., 2021). In turn, taVNS likely modulates other transmitter systems as well, given the various projections from the NTS to other core areas (Krahl and Clark, 2012; Frangos et al., 2015). Although previous studies have examined the effects of noninvasive taVNS on PD as well, the results have been inconsistent (Keute et al., 2019; D'Agostini et al., 2022), potentially because of nonoptimal stimulation parameters (Ludwig et al., 2021). These former studies often applied tonic 30 s on/30 s off stimulation (Burger et al., 2020a; D'Agostini et al., 2022), which might be ineffective in reliably modulating the PD. However, more promising results were demonstrated with short bursts (600–5000 ms) of taVNS (Sharon et al., 2021; Urbin et al., 2021; Villani et al., 2022; D'Agostini et al., 2023), but these effects on PD have largely been investigated in the absence of a specific behavioral task, that is, in response to taVNS itself (Sharon et al., 2021; Urbin et al., 2021; D'Agostini et al., 2023) or stimulation was applied before stimulus onset (Villani et al., 2022). Furthermore, studies have largely ignored the pupil light reflex (PLR), which is also influenced by NA activity (Bitsios et al., 1999; Hysek and Liechti, 2012). The PLR is the rapid constriction of the pupil in response to light and is controlled by the brainstem Edinger–Westphal nucleus (EWN; Hall and Chilcott, 2018).

In addition to pupillometry, cortical oscillations have been indicated as noradrenergic markers, including alpha oscillations as a marker for cortical arousal (Dahl et al., 2022) and frontal-midline (FM) theta power (Dippel et al., 2017) as an electrophysiological correlate of cognitive control (Cavanagh and Frank, 2014). In line with this, taVNS has been shown to decrease occipital alpha power at rest (Sharon et al., 2021) and to increase FM theta power during a cognitive control task (Keute et al., 2020).

In this study, we investigated how phasic, event-related taVNS modulates pupillary and electrophysiological markers of LC-NA activation during a response conflict task. We expected increased PD and FM theta power, as well as reduced alpha power during cognitive processing, and reduced pupil constriction and alpha power during the PLR following taVNS.

## Materials and Methods

### Subjects

Twenty-nine subjects (16 female) ranging in age from 18 to 40 years (mean = 26.5; SD = 6) participated in this study. All subjects provided written informed consent, reported no history of neurologic or psychiatric disease, and reported normal or corrected-to-normal vision using contact lenses. Recordings took place at Otto-von-Guericke University, Magdeburg, Germany, and were approved by the Ethical Committee of the Otto-von-Guericke University Magdeburg. For participation, subjects were reimbursed with money or course credit.

### Experimental procedure

After providing written informed consent, subjects were prepared for the MEG recording. Electrodes for the vertical and horizontal EOG were attached above and below the right eye as well as on both outer eye corners (canthus). Impedance for EOG electrodes was kept below 10 kΩ. Head shape was then digitized using a Polhemus Fastrak motion tracker. The coordinates of three anatomic landmarks (nasion, left and right preauricular point), the five head position indicator coils, and a minimum of 200 additional points on the scalp were digitized. The stimulation electrodes (see below) were then attached, and the subjects were comfortably seated in the MEG chamber.

**Figure 1.**
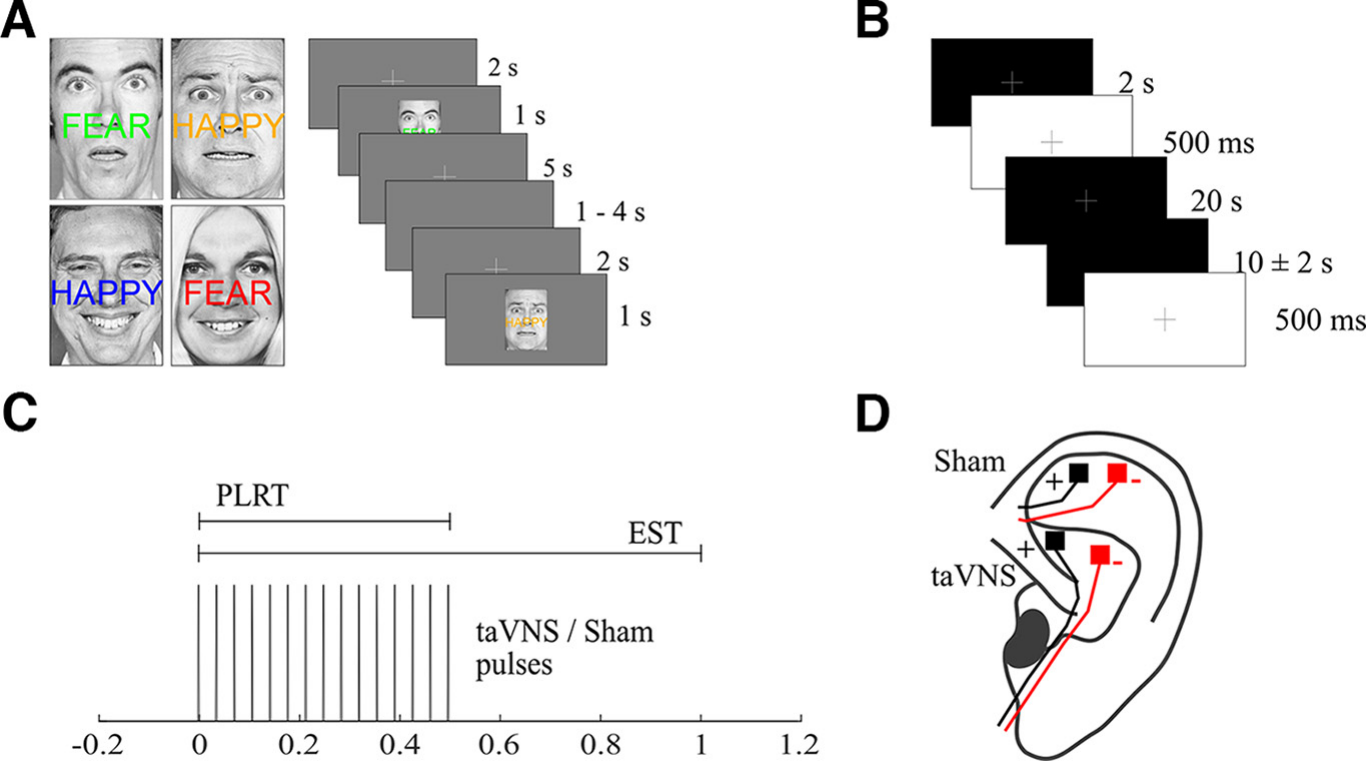
Paradigm. ***A***, Subjects performed the EST, that is, categorizing the emotional expression of faces while ignoring the overlaid word. Each trial was either congruent or incongruent. ***B***, Subjects also performed a PLRT, where they were exposed to short, bright light stimuli. ***C***, Coinciding with each stimulus onset, either taVNS or sham stimulation was applied as 500 ms trains at 30 Hz. Pulse width of the monophasic square wave pulses was 200 μs. Top, Horizontal bars show the stimulus duration during the EST and PLRT. ***D***, taVNS was applied to the cymba concha region of the left ear while sham stimulation was applied to the scapha.

We applied two different tasks during the same recording session: The emotional Stroop task (EST) (Grueschow et al., 2020, 2021, 2022) and a passive Pupillary Light Reflex Task (PLRT). Both tasks were performed twice by each subject, once during active taVNS and once during sham stimulation. The order of stimulation was counterbalanced across subjects (i.e., half received taVNS in the first half of the experiment and sham stimulation in the second half of the experiment or vice versa). Subjects first performed six blocks of the EST during which they were asked to categorize the emotional expression of faces (happy or fearful) while ignoring an overlaid emotional word (happy or fear). Trials were either congruent (word matches the facial expression) or incongruent (word does not match the facial expression; Fig. 1*A*). The color of the overlaid word was randomly chosen for each trial to avoid adaptation. Each block consisted of 20 pictures (10 congruent, 10 incongruent). Each trial started with the presentation of a gray fixation cross in the center of the screen for 2 s. After that, the fixation cross disappeared and the actual stimulus picture was presented for 1 s, after which only the fixation cross was again visible for 5 s. The fixation cross then disappeared for a variable interstimulus interval (ISI) between 1 and 4 s. Afterward, a new trial started with the presentation of the fixation cross for 2 s. Subjects were instructed to fixate the cross whenever visible on screen to avoid eye movement and to avoid blinking as much as possible. They were further instructed that they could blink during periods with no fixation cross on screen, that is, during the ISI. Pictures subtended a visual angle of ∼8 × 11.4°, which does not warrant the use of saccades to identify the emotional content. The overlaid word was centered at the same *x* and *y* coordinates as the fixation cross to further reduce the potential for saccades. All face stimuli were equally distributed between congruent and incongruent conditions. Hence, the net luminance for both conditions was identical. Responses were given via button press with the left or right index finger. The assignment of which finger had to be pressed in response to which face was counterbalanced across subjects (i.e., half the subjects had to press the left index finger following a happy face and right index finger for fearful faces and vice versa in the other half of subjects). Parallel with stimulus onset, subjects received either taVNS or sham stimulation (see below). Following completion of the EST, subjects performed the PLRT (Fig. 1*B*). This part consisted of one block of 20 trials with passive bright light stimulation to elicit a PLR. Subjects were first dark adapted for 2 min (∼0.14 cd/m^2^) before the actual PLRT started. Each trial started with the presentation of a gray fixation cross in the center of the screen for 2 s, after which the screen turned white for 500 ms (∼179 cd/m^2^). Afterward, only the fixation cross remained on screen for 20 s followed by a variable ISI between 8 and 12 s. Subjects were again instructed to fixate the cross whenever visible on screen to avoid eye movements and blinking as much as possible. No button presses were required in this task. Again, parallel with stimulus onset, subjects received either taVNS or sham stimulation. Subsequently, both tasks were then completed again with the remaining stimulation condition. Subjects were instructed that they could take a self-paced break after each experimental block. All stimuli were presented from the experimental computer running MATLAB R2018b (MathWorks) and Psychtoolbox 3 (Brainard, 1997; Pelli, 1997; Kleiner et al., 2007).

### Electrical stimulation

Stimulation was delivered as 500 ms long trains of monophasic square wave pulses with a frequency of 30 Hz. Each pulse had a width of 200 μs (Fig. 1*C*). The stimulation amplitude was set to 2 mA in line with previous studies (Capone et al., 2021; Sharon et al., 2021). We applied taVNS and sham stimulation in the same session using two Digitimer DS7A constant current stimulators outside the magnetically shielded room and two pairs of medical Ag/AgCl electrodes (Ambu Neuroline 700) cut to a size of 4 × 4 mm. Both Digitimers were connected to the experimental computer via a DATAPixx processing unit (Vpixx Technology). For taVNS, electrodes were attached to the cymba conchae and for sham stimulation to the scapha of the left ear (Fig. 1*D*). Although most taVNS studies use the ear lobe as the location for the sham stimulation, the scapha has been proposed as more suitable for sham stimulation (Cakmak, 2019). In both cases, the anode was placed more rostrally. Before electrode placement, both target regions were cleaned with disinfectant alcohol. A small amount of Ten20 paste (Weaver and Company) was used to ensure proper conductance. Stimulation was tested before the start of the experiment by applying single trains of 500 ms at 2 mA to ensure that stimulation worked properly and to determine whether stimulation felt uncomfortable. If the subjects reported pain or an unpleasant sensation during this test, the stimulation intensity in the respective condition was reduced in 0.1 mA steps until subjects no longer reported discomfort during stimulation. This resulted in an average stimulation intensity of 1.91 mA for taVNS (SD = 0.23) and 1.87 mA for sham (SD = 0.25). There was no statistical difference between these conditions (*t*_(28)_ = 0.87, *p* = 0.39). Of the 29 subjects, 17 rated the 2 mA in both stimulation conditions as not uncomfortable. Ten subjects rated the initial 2 mA in one stimulation condition as uncomfortable, and only two subjects rated 2 mA in both conditions as uncomfortable.

### Data acquisition

Pupil diameter was recorded monocularly from the left eye using a MEG compatible EyeLink 1000 long-range mount (SR Research). The infrared camera was fixated below the video screen inside the MEG chamber. The sampling rate was set to 500 Hz. At the start of the experiment and after the first half, the camera was calibrated using the built in five-point calibration. The entire recording session lasted ∼65 min.

Brain activity throughout the task was recorded in a sitting position using a whole-head Elekta Neuromag TRIUX system in a magnetically shielded room (VacuumSchmelze). The sampling rate was set to 1000 Hz. An online bandpass filter was applied between 0.1 and 330 Hz. Visual stimuli were presented via rear projection using an LCD projector (DLA-G150CLE, JVC) outside the recording booth. The semitransparent screen was placed 100 cm in front of the subjects. Responses were given using a MEG-compatible LUMItouch response system (Photon Control).

### Data preprocessing

#### Pupillometry

Off-line preprocessing of pupil data was performed using custom scripts for MATLAB R2018b software following the recommendations from Kret and Sjak-Shie (2019). Continuous data were cut into epochs from −2 s to 5 s around stimulus onset for the EST and from −2 to 7 s for the PLRT. Trials with >50% missing data points were rejected. Subjects with >50% excluded trials in one condition were wholly excluded from statistical analyses (*N* = 5). For the remaining trials, the normalized dilation speed time series was computed to detect blinks and other artifacts. These manifest as disproportionally large changes in pupil dilation relative to the adjacent samples (Kret and Sjak-Shie, 2019). To detect dilation speed outliers, the median absolute deviation (MAD) was calculated from the normalized dilation speed time series. A threshold was then calculated by multiplying the MAD with a constant and adding the median of the normalized dilation speed time series (Kret and Sjak-Shie, 2019). This constant was set to 2.5 (Leys et al., 2013). In the first step, data points whose dilation speed exceeded this threshold were removed. To detect remaining outliers that resisted this first rejection, a smoothed trend line was then formed by linearly interpolating the resulting gaps in the pupil time series and smoothing with a 100 ms moving average. Remaining outliers in the pupil time series from this trend line were then detected as in the first step. The missing data points in the cleaned time series were then linearly interpolated. Pupil data were then *z*-scored, baseline corrected to the average of the 200 ms before stimulus onset, and downsampled to 100 Hz.

#### MEG

Off-line preprocessing of MEG data was performed using MATLAB R2018b and the FieldTrip toolbox (Oostenveld et al., 2011). In the first step, the stimulation artifact had to be cleaned from the data. Sole filtering or independent component analysis (ICA) approaches are not sufficient to sufficiently clean the stimulation artifact from the data because of nonlinear effects caused by physiological processes such as respiration and heartbeat. Thus, an autoregressive interpolation method as in Keatch et al. (2022) was used. The DATAPixx processing unit provided event markers each time a stimulation pulse was triggered. Ten milliseconds after each marker were excluded and replaced with NaNs (Not a Number). Missing data were then interpolated using the built-in MATLAB function fillgaps() with a prediction sequence length of 25 ms before and after each gap to estimate the missing values. The data cleaned in this way were subsequently high-pass filtered at 1 Hz and low-pass filtered at 60 Hz. A band-stop filter (49–51 Hz) was applied to suppress line noise. All filters used were bidirectional infinite impulse response Butterworth filters. Continuous data were then segmented into epochs from −2 s to 5 s relative to stimulus onset. Remaining artifacts like blinks and heartbeats were removed by applying an ICA (Infomax algorithm) to the data. Components reflecting remaining artifactual activity were excluded after visual inspection. Epochs were then downsampled to 250 Hz and baseline corrected relative to the 200 ms before stimulus onset. Average baseline values were retained for subsequent analysis to check for potential baseline differences in brain activity between conditions. Given the greater amount of interpolation necessary, we calculated spectral power using fast Fourier transformation (FFT) from a 500-ms-long Hanning tapered window directly after stimulus onset. The rationale for choosing this time window was based on the previous literature on cognitive control and theta activity, showing that an effect of conflict processing on FM theta develops within the first 500 ms after stimulus presentation (Cavanagh and Frank, 2014). We further decided to restrict this part of the analysis on the first 500 ms to avoid comparing interpolated with noninterpolated data. The advantage of the FFT approach is that it only uses the selected time window compared with a moving window method like Morlet wavelets that would incorporate activity from adjacent time points where no interpolation was applied. Frequencies were set from 2 to 14 Hz in 0.5 Hz steps.

To further investigate the effects of taVNS on longer lasting effects in the alpha range at occipital sites (Sharon et al., 2021), we also computed time-frequency representations (TFR) of these sensors using Morlet wavelets. TFRs were computed for the whole trial between −1000 and 5000 ms relative to stimulus onset in 40 ms time bins for frequencies between 2 and 14 Hz in 0.5 Hz steps. The number of cycles for the wavelet was set to five. A baseline correction using decibel transformation was applied relative to the time period from −500 to −200 ms before stimulus onset.

### Statistical analyses

Statistical analyses were performed using R 4.1.2 software (https://www.R-project.org/; http://www.rstudio.com/). Linear mixed effect models (LMMs) were constructed using the lme4 package (Bates et al., 2015) in R and fitted using the restricted maximum likelihood method. Significance values for the individual predictors were obtained with the Anova() function from the car package (Fox and Weisberg, 2019) performing likelihood-ratio chi-square tests. Significant interactions between predictors were resolved by running additional LMMs with one of the predictors nested within the other. This allowed us to calculate the effect of one predictor at both levels of the other (Frömer et al., 2018). As nested models are not supported by the Anova() function, significance was determined by estimating the degrees of freedom for the *t* values using the Satterthwaithe approximation performed in the lmerTest package (Kuznetsova et al., 2017). Cluster-based permutation testing (CBPT) was used to analyze time series and Fourier spectra between different conditions. CBPT is a standard, data driven approach to resolve the multiple comparison problem (Maris and Oostenveld, 2007). In the first step, statistical tests are performed at each time point. Adjacent time points that exceed a (uncorrected) statistical threshold are clustered. Test statistics are summed to form the empirical cluster size. In the second step, a null hypothesis distribution is formed. For that, condition labels are randomly shuffled, and the statistical tests are repeated at each time point. Again, adjacent time points exceeding the threshold level are clustered, and test statistics in the largest cluster are summed to obtain one cluster size per iteration. This shuffling and calculating of test statistics is repeated for a certain number of iterations. The empirical cluster size from the first step is then thresholded against the distribution of randomly permuted cluster sizes to obtain the *p* value of clusters. CBPT was performed using the permutes package (https://CRAN.R-project.org/package=permutes). In each permutation analysis 10,000 permutations were used to form the null hypothesis distribution. We report average and maximum effect size (Cohen's *d*) from each significant cluster (Meyer et al., 2021).

#### Behavior

Because of technical difficulties, behavioral responses from three subjects were not recorded, Thus, behavioral results are based on 26 subjects. A mixed effect logistic regression on the single-trial level was used to determine whether stimulation (taVNS vs sham), congruency (congruent vs incongruent trials), or the interaction between these two predictors affected performance. Trials were coded as one when a correct response was given in the respective trial and as zero otherwise (i.e., incorrect answers and omissions). Similarly, sham stimulation was coded as zero and taVNS as one. The face emotion (fearful vs happy) was included as covariate in the model. The trial number was also included as predictor to account for possible effects of time spent on task. Finally, to control for the small difference in stimulation intensity between taVNS and sham stimulation, we included stimulation intensity as a continuous variable in the analysis. The random effect structure contained random intercepts and slopes for stimulation and congruency across subjects as follows:

hits∼trialnumber+face+intensity+stimulation*congruency+(1+stimulation+congruency|subjectID).

For the reaction times (RTs), an LMM was fitted on the single-trial level with RT as outcome variable and stimulation, congruency and their interaction as fixed effect. The trial number, face emotion-condition and stimulation intensity were again included as covariates. The random effect structure contained random intercepts and slopes for stimulation and congruency across subjects as follows:

RT∼trialnumber+face+intensity+stimulation*congruency+(1+stimulation+congruency|subjectID).

#### Pupillometry

Five subjects were excluded from the analysis of pupil data because of an excessive number of excluded trials. In the remaining 24 subjects, an average of 3.57 trials were excluded (range: 0, 28; SD = 6). The number of excluded trials did not differ between conditions (*F*_(3,69)_ = 0.42, *p* = 0.74). For the EST, average pupil time series between −500 and 5000 ms were analyzed to determine whether pupil diameters differed between conditions. LMMs were fitted at each time point with average pupil diameter as outcome. Congruency (dummy coded, 0 = incongruent, 1 = congruent), stimulation (dummy coded, 0 = sham, 1 = taVNS), and their interaction were set as fixed effects. The continuous predictor stimulation intensity was again included as a covariate. Random intercepts and slopes for congruency and stimulation across subjects were set as random effects as in the following:

pupil ∼ intensity+ stimulation * congruency+(1+stimulation+congruency | subjectID )

For the PLRT we examined in a similar fashion the effect of the predictors stimulation (dummy coded, 0 = sham, 1 = taVNS) and stimulation intensity as fixed effect on pupil diameter at each time point between −500 and 7000 ms relative to stimulus onset. The random effect structure contained random intercepts and slopes for stimulation across subjects as follows:

pupil ∼ intensity+ stimulation+(1+stimulation |subjectID).

Apart from the pupil diameter at each time point, previous pharmacological studies have shown that other dynamics of the pupil light reflex are also affected by an increase in NA, among others. These include the onset latency of the pupil light reflex, the velocity with which the pupil constricts and re-dilates, as well as the maximal constriction amplitude (Theofilopoulos et al., 1995; Bitsios et al., 1999; Hysek and Liechti, 2012). To further elucidate the effect of taVNS on dynamics of the PLR, we thus extracted the following parameters from the preprocessed and averaged PLR time series for each subject from both stimulation conditions: The peak constriction amplitude was determined as the maximal negative value in the PLR time series after stimulus onset. To determine velocity-based parameters of pupillary dynamics, we then computed the first (velocity) and second (acceleration) derivative of the PLR time series. The onset of the pupil constriction was determined as the most negative acceleration in the first period of the second derivative (Bergamin and Kardon, 2003). We then determined the peak and average constriction velocity after stimulus onset as well as the peak and average redilation velocity after the maximum of the pupil constriction. During constriction of the pupil after light onset, when the velocity is negative, the peak constriction velocity was determined as the peak negative velocity between onset of the PLR and the peak constriction amplitude. Similarly, the average constriction velocity was determined as the mean velocity between PLR onset and the maximal pupil constriction. Finally, during redilation of the pupil, when velocity is positive, the peak dilation velocity was determined as peak positive velocity after the maximal pupil constriction. The average dilation velocity was calculated as mean velocity after the maximal pupil constriction. These parameters were again analyzed using separate LMMs. The models for all dependent variables (DV) contained the predictor stimulation as well as the stimulation intensity as fixed effect and random intercepts across subjects as follows:

DV ∼ intensity + stimulation+(1 | subjectID).

#### MEG

Seven subjects were excluded from analysis of the MEG data after visual inspection of the data from each trial and sensor. Exclusion criteria were flat or severely noise-corrupted sensors in the frontal-midline or occipital-midline region of interest (ROI). Average baseline brain activity from frontal and occipital ROIs before stimulus onset was analyzed between conditions (e.g., PLR during taVNS, congruent trials during taVNS, …) using two LMMs. Both models contained the predictor condition as fixed effect as well as random intercepts across subjects as follows:

$$Baseline_{frontal/occipital}∼condition+\text{(}1\text{|}subjectID).$$

Spectral power from the remaining 22 datasets was analyzed using CBPT. For the EST, Fourier spectra from FM and occipital-midline (OM) gradiometers were first averaged and then subjected to separate permutation analysis. LMMs were fitted at each frequency point between 2 and 14 Hz with power as outcome and congruency, stimulation, and their interaction as well stimulation intensity as fixed effects. The random effect structure contained random intercepts and slopes for the congruency and stimulation across subjects as follows:

$$PSD_{frontal/occipital}∼intensity+stimulation*congruency+\text{(}1+congruency+stimulation\text{|}subjectID).$$

For the PLRT, spectra from OM and FM gradiometers were first averaged and then analyzed in the same way with power as outcome and stimulation intensity and stimulation as fixed effect. Random slopes and intercepts for stimulation across subjects were sat as random effect as follows:

$$PSD_{frontal/occipital}∼intensity+stimulation+\text{(}1+stimulation\text{|}subjectID).$$

Differences between taVNS and sham stimulation in the TFRs of OM gradiometers were also analyzed using CBPT. To avoid comparing interpolated with noninterpolated time periods, we restricted the analysis to time period from 600 to 5000 ms poststimulus, that is, after the offset of stimulation where contamination from an electrical artifact can be ruled out. The CBPT was then performed using dependent sample *t* tests as implemented in FieldTrip (Oostenveld et al., 2011) again with 10,000 permutations.

#### Relationship between parameters across tasks

We further investigated the relationship between taVNS-specific effects on electrophysiological and pupillometric measures between the two tasks. First, for the EST and PLRT we calculated stimulation-related changes, that is, deltas (taVNS minus sham difference) in FM and OM power spectra (ΔPSD_frontal_, ΔPSD_occipital_), the pupil diameter time series (ΔPD_EST,_ ΔPD_PLRT_), the average power difference at FM and OM sensors from significant clusters during the EST and PLRT (Δfm.est, Δom.est, Δfm.plrt, Δom.plrt1, Δom.plrt2), and the average pupil diameter difference from significant clusters indicated by the CBPT (Δpupil.est, Δpupil.plrt1, Δpupil.plrt2). Second, we analyzed whether averaged FM and OM power changes from clusters derived via CBPT in one task were predictive of FM and OM power changes in the other task. That is, at FM sensors during the EST, we analyzed whether ΔPSD_frontal_ could be predicted by Δfm.plrt. Using CBPT we tested the following model at each frequency between 2 and 14 Hz:

$$\Delta PSD_{frontal}∼\Delta fm.plrt+\text{(}1\text{|}subjectID).$$

Vice versa at FM sensors during the PLRT, we analyzed whether the respective ΔPSD_frontal_ could be predicted by the average power difference from the FM stimulation cluster during the EST using CBPT with the following model at each frequency between 2 and 14 Hz:

$$\Delta PSD_{frontal}∼\Delta fm.est+\text{(}1\text{|}subjectID).$$

At OM sensors during the EST we tested whether ΔPSD_occipital_ could be predicted by Δom.plrt1 and Δom.plrt2 again using CBPT with the following model at each time point between 2 and 14 Hz:

$$\Delta PSD_{occipital}∼\Delta om.plrt1+\Delta om.plrt2+\text{(}1\text{|}subjectID).$$

Vice versa at OM sensors during the PLRT, we tested whether the respective ΔPSD_occipital_ could be predicted by Δom.est in the same way with the following model at each frequency between 2 and 14 Hz:

$$\Delta PSD_{occipital}∼\Delta om.est+\text{(}1\text{|}subjectID).$$

We finally tested whether stimulation-specific changes in PD during one task could be predicted by the averaged difference in PD from significant clusters in the other. That is, for EST we tested whether ΔPD_EST_ could be predicted by Δpupil.plrt1 and Δpupil.plrt2. CBPT was used to test the following model at each time point between −200 and 5000 ms:

$$\Delta PD_{EST}∼\Delta pupil.plrt1+\Delta pupil.plrt2+\text{(}1\text{|}subjectID).$$

Vice versa for the PLRT, we tested whether ΔPD_PLRT_ could be predicted by Δpupil.est with the following model at each time point between −200 and 7000 ms:

$$\Delta PD_{PLRT}∼\Delta pupil.est+\text{(}1\text{|}subjectID).$$

## Results

### Behavior

**Figure 2.**
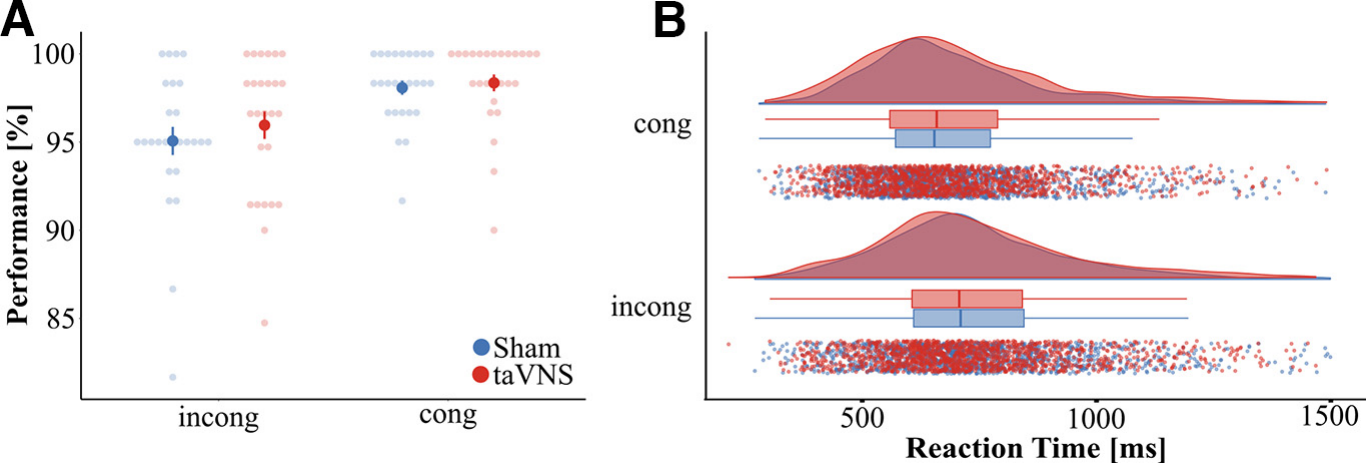
Behavior. ***A***, During the EST, subjects made fewer errors in congruent trials and during taVNS. ***B***, Subjects responded faster during congruent trials compared with incongruent trials, but no effect of stimulation and no stimulation-congruency-interaction was observed. Error bars in ***A*** represent SEM.

Accuracy in the EST was systematically modulated by the congruency of the stimuli and taVNS. The mixed effect logistic regression revealed that the predictor congruency was a significant indicator of accuracy (β = 0.92, *p* < 0.001, Fig. 2*A*). This positive beta estimate corresponds to an odds ratio (OR) of 2.51, indicating that in congruent trials subjects were ∼2.51 times more likely to give a correct response. Importantly, we also observed a significant effect of vagus nerve stimulation (β = 0.44, *p* = 0.03) corresponding to an OR of 1.55. This indicates that during taVNS, subjects were 1.55 times more likely to give a correct response than in the sham stimulation. Furthermore, we observed a significant effect of the predictor trial number (β = 0.005, *p* < 0.001), corresponding to an OR of 1.005, which indicates that accuracy slowly improved over the duration of the experiment. No significant effect was observed for the predictor face emotion (β = 0.06, *p* = 0.7), stimulation intensity (β = −0.24, *p* = 0.57), or the stimulation-by-congruency interaction (β = 0.02, *p* = 0.95).

Reaction time during the EST was significantly modulated by congruency. The LMM revealed a significant effect of the predictor congruency (χ_(1)_^2^ = 57.13, *p* < 0.001; Fig. 2*B*). Subjects responded faster during congruent trials (mean = 699.88 ms, SD = 260.62 ms) compared with incongruent trials (mean = 753.69 ms, SD = 254.43 ms). Further, the trial number was also a significant indicator of RT (χ_(1)_^2^ = 75.59, *p* < 0.001), indicating that subjects responded faster over the duration of the experiment (β = −0.49, SE = 0.06, *t* = −8.6). No effects on RTs were observed for the predictors stimulation (χ_(1)_^2^ = 0.37, *p* = 0.54), face (χ_(1)_^2^ = 2.01, *p* = 0.16), stimulation intensity (χ_(1)_^2^ = 0.43, *p* = 0.51), or the stimulation-by-congruency interaction (χ_(1)_^2^ = 1.36, *p* = 0.24).

### Pupillometry

**Figure 3.**
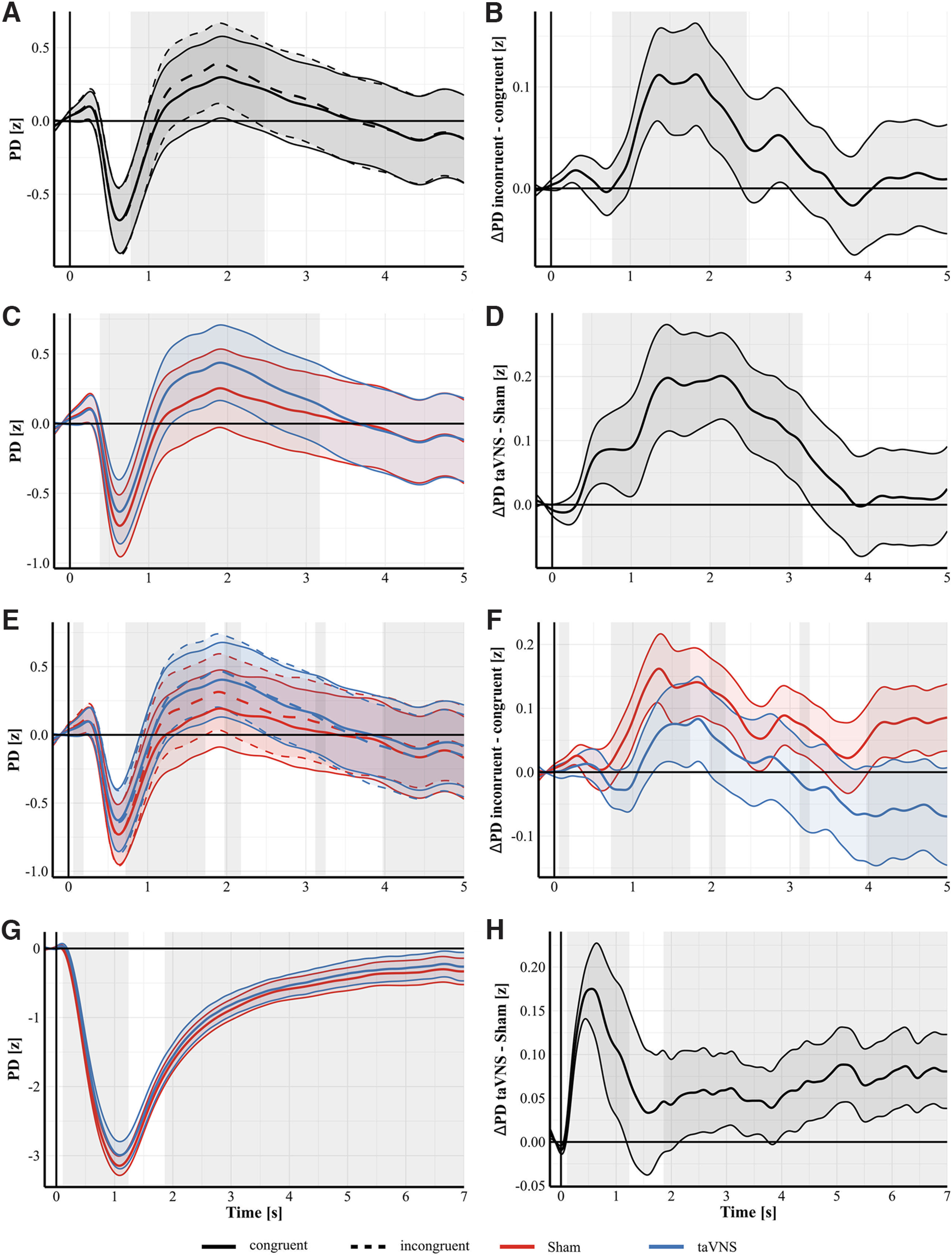
Pupil dilation separately for conditions (left) and difference waves (right). ***A***, ***B***, Effect of congruency. Grand average PD from the EST during incongruent (dashed lines) and congruent (solid lines) trials (***A***). Difference between incongruent and congruent trials during the EST (***B***). Permutation testing indicated a significant difference between incongruent and congruent trials corresponding to a cluster between 770 and 2500 ms poststimulus with higher PD during incongruent trials compared with congruent trials (gray shaded rectangle indicates the significant cluster). ***C***, ***D***, Effect of stimulation. Grand average PD from the EST during taVNS (blue) and Sham stimulation (red; ***C***). Difference between taVNS and Sham stimulation during the EST (***D***). Permutation testing indicated a significant difference corresponding to cluster between 400 and 3200 ms poststimulus (gray shaded rectangle) with higher PD during taVNS. ***E***, ***F***, Stimulation-by-congruency interaction. Grand average PD from the EST for both congruent (solid) and incongruent (dashed) trials during taVNS (blue) and Sham (red) stimulation (***E***). Difference between incongruent and congruent trials during taVNS (blue) and Sham stimulation (red; ***F***). Permutation testing indicated a significant stimulation-by-congruency interaction corresponding to five clusters in our data (gray shaded rectangles; see Pupillometry Results for details). ***G***, ***H***, Effect of stimulation during the PLRT. Grand average PD from the PLRT during taVNS (blue) and Sham stimulation (red; ***G***). Difference between taVNS (blue) and Sham stimulation (red) during the PLRT (***H***). Permutation testing indicated a significant difference between taVNS and Sham stimulation corresponding to two clusters between 100 and 1200 ms and 1900 and 7000 ms poststimulus (gray shaded rectangles). PD was higher (i.e., less constricted) during taVNS compared with Sham stimulation in both clusters. Shaded areas around curves indicate SEM.

Figure 3 shows the PD and differences in pupil dilation separately for each condition during the EST and PLRT. PD during the EST was systematically modulated by congruency and stimulation. For the factor congruency, regardless of stimulation, CBPT indicated a significant difference between conditions. This corresponded to a significant cluster in the observed data between 770 and 2500 ms poststimulus (*p_cluster_* = 0.0001; Fig. 3*A*,*B*) with greater pupil dilation during incongruent trials (mean = 0.19 *z* score, SD = 1.37) compared with congruent trials (mean = 0.12 *z* score, SD = 1.38). Cohen's *d* of the average pupil dilations from that cluster (*d_avg_*) was 0.43, the maximal Cohen's *d* during that cluster (*d_max_*) was 0.58 at 1280 ms poststimulus. Both congruency conditions show the well-known light reflex to the face stimuli (Grueschow et al., 2021, 2022; Fig. 3*A*), which is absent when both conditions are directly contrasted (Fig. 3*B*). The CBPT further indicated a significant effect of the predictor stimulation, corresponding to a cluster between 400 and 3200 ms poststimulus (*p_cluster_* = 0.0001; Fig. 3*C*,*D*) with greater pupil dilation during taVNS (mean = 0.13 *z* score, SD = 1.35) compared with sham stimulation (mean = −0.005 *z* score, SD = 1.37, *d_avg_* = −0.47, *d_max_* = −0.6 at 2150 ms poststimulus). Furthermore, the CBPT also indicated a congruency-by-stimulation interaction corresponding to five clusters between 60 and 190 ms, 720 and 1730 ms, 1970 and 2180 ms, 3120 and 3250 ms, and 3970 and 5000 ms poststimulus (Fig. 3*E*,*F*). Figure 3*F* shows the difference between incongruent and congruent trials for both, sham and taVNS. For all clusters, nested LMMs were computed to resolve these interactions. However, only the model with stimulation nested in congruency for the cluster between 720 and 1730 ms indicated a trend toward a taVNS effect only during congruent trials (β = 0.13, SE = 0.07, *t*_(69)_ = 1.9, *p* = 0.06) but not during incongruent trials (β = 0.07, SE = 0.07, *t*_(69)_ = 1, *p* = 0.33). No other nested model indicated significant effects (all *p* values > 0.16). Finally, the CBPT indicated an effect of the factor stimulation intensity corresponding to a cluster between 860 and 5000 ms poststimulus (*p_cluster_* = 0.0001). As stimulation intensity is a continuous variable, we report the average beta estimate from the significant cluster to provide an overview of the size and direction of the effect. This resulted in an average beta of 0.236, indicating that higher stimulation intensities were associated with higher PD.

Pupil constriction during the PLRT was also systematically modulated by stimulation and stimulation intensity. The CBPT indicated a significant effect of the continuous variable stimulation intensity corresponding to a cluster between 300 and 6500 ms poststimulus (*p_cluster_* = 0.0001). Here, the average beta estimate was −0.299, indicating that an increase in stimulation intensity reduced the pupil constriction. The CBPT also indicated a difference between taVNS and sham stimulation corresponding to two clusters, the first between 100 and 1200 ms and the second between 1900 and 7000 ms poststimulus (both *p_cluster_* = 0.0001; Fig. 3*G*,*H*). Average pupil diameter in both clusters was higher (i.e., less constricted) during taVNS (cluster 1, mean = −1.85 z score, SD, = 1.32; cluster 2, mean = −0.59 z score, SD = 0.93) compared with sham (cluster 1, mean = −2 z score, SD = 1.24, *d_avg_* = −0.51, *d_max_* = −1.47 at 340 ms; cluster 2, mean = −0.66 z score, SD = 0.89, *d_avg_* = −0.35, *d_max_* = −0.42 at 5180 ms). In cluster 1, the peak constriction amplitude (χ_(1)_^2^ = 7.56, *p* = 0.006), the peak constriction velocity (χ_(1)_^2^ = 12.03, *p* = 0.0006), and the average constriction velocity (χ_(1)_^2^ = 11.44, *p* = 0.0007) were modulated by stimulation intensity but not by stimulation (all *p* values > 0.09). Inspection of the corresponding beta estimates indicated that higher stimulation intensities corresponded to higher (i.e., more negative) peak constriction amplitudes (β = −0.59, SE = 0.21, *t* = −2.75), higher peak constriction velocities (β = −1.52, SE = 0.44, *t* = −3.47), and higher average constriction amplitudes (β = −0.82, SE = 0.24, *t* = −3.38). The onset of the PLR was significantly modulated by stimulation (χ_(1)_^2^ = 9.92, *p* = 0.002) but not by stimulation intensity (*p* = 0.15). The onset of the PLR was delayed during taVNS (mean = 197.92 ms, SD = 66.59) compared with sham stimulation (mean = 170.83 ms, SD = 62.27, β = 25.3, SE = 8.03, *t* = 3.15). In the second cluster, neither average nor peak dilation velocity were affected by stimulation (all *p* values > 0.35) or by intensity (all *p* values > 0.05).

### MEG

**Figure 4.**
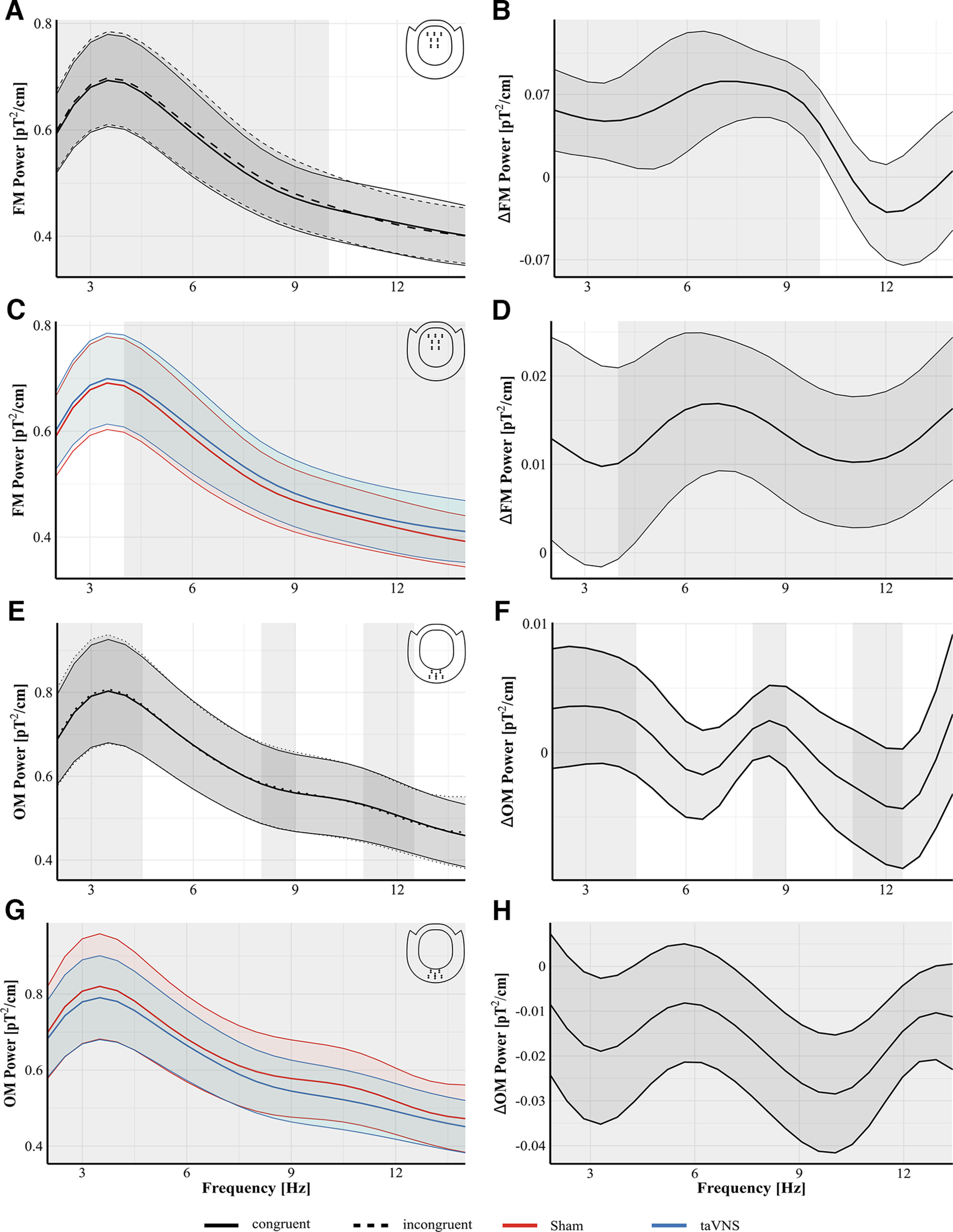
Fourier spectra for conditions (left) and difference waves (right) during the EST. ***A***, ***B***, Effect of congruency on FM sensors. Inset, top right (***A***), Grand average FFT at FM sensors from the EST during incongruent (dashed) and congruent (solid) trials. Difference between incongruent and congruent trials during the EST (***B***). Permutation testing indicated a significant difference between incongruent and congruent trials corresponding to a cluster between 2 and 10 Hz with higher power during incongruent trials compared with congruent trials (gray shaded rectangle indicates the significant cluster). ***C***, ***D***, Effect of stimulation on FM sensors. Inset, top right (***C***), Grand average FFT at FM sensors from the EST during taVNS (blue) and Sham stimulation (red). Difference between taVNS and Sham stimulation during the EST (***D***). Permutation testing indicated a significant difference corresponding to cluster between 4 and 14 Hz (gray shaded rectangle) with higher power during taVNS. ***E***, ***F***, Effect of congruency on OM sensors in the EST. Inset, top right (***E***), Grand average FFT at OM sensors for both congruent (solid) and incongruent (dashed) trials. Difference between incongruent and congruent trials during the EST (***F***). Permutation testing indicated a significant difference corresponding to three clusters (gray shaded rectangles; see MEG Results for details). ***G***, ***H***, Effect of stimulation on OM sensors in the EST. Inset, top right (***G***), Grand average FFT at OM sensors from the EST during taVNS (blue) and Sham stimulation (red). Difference between taVNS and Sham stimulation during the EST (***H***). Permutation testing indicated a significant difference corresponding to cluster between 2 and 14 Hz (gray shaded rectangle) with lower power during taVNS. Shaded areas around curves indicate SEM.

Baseline brain activity at frontal and occipital sensors between conditions was analyzed using separate LMMs. Neither model revealed an effect of condition (frontal, χ_(5)_^2^ = 6.8, *p* = 0.23; occipital, χ_(5)_^2^ = 0.51, *p* = 0.99) indicating there were no differences in baseline brain activity. Figure 4 shows the power at FM and OM gradiometers during the EST for the different experimental conditions and their respective difference. Power at FM gradiometers during the EST was systematically modulated by congruency, stimulation, and stimulation intensity. The CBPT indicated a significant effect of the predictor stimulation intensity corresponding to a cluster between 2 and 14 Hz (*p_cluster_* = 0.0001). The average beta estimate from that cluster was −4^−26^, indicating a reduction in power with increasing stimulation intensity. The CBPT further indicated an effect of the predictor congruency corresponding to a cluster between 2 and 10 Hz (*p_cluster_* = 0.0001; Fig. 4*A*,*B*). FM power within that frequency range was higher during incongruent trials (mean = 0.59 pT^2^/cm, SD = 0.4) compared with congruent trials (mean = 0.58 pT^2^/cm, SD = 0.39, *d_avg_* = 0.47, *d_max_* = 0.62 at 8.5 Hz). The CBPT also indicated an effect of the predictor stimulation corresponding to a cluster between 4 and 14 Hz (*p_cluster_* = 0.0001; Fig. 4*C*,*D*). FM power within that frequency range was higher during taVNS (mean = 0.522 pT^2^/cm, SD = 0.39) compared with sham stimulation (mean = 0.508 pT^2^/cm, SD = 0.38, *d_avg_* = −0.4, *d_max_* = 0.52 at 7.5 Hz). Finally, the CBPT indicated a congruency-by-stimulation interaction on FM power during the EST corresponding to two clusters in our data, one between 2 and 4.5 Hz (*p_cluster_* = 0.0001) and the second between 8.5 and 10 Hz (*p_cluster_* = 0.0003). Nested linear mixed models were used to resolve the interaction in both clusters. For the first cluster between 2 and 4.5 Hz, neither the model with congruency nested in stimulation nor the respective model with stimulation nested in congruency revealed significant effects (all *p* values > 0.06). In the second cluster between 8.5 and 10 Hz, the model with stimulation nested in congruency revealed a significant effect of stimulation only during incongruent trials (β = 1.73^−26^, SE = 6.7^−27^, *t* = 2.6, *p* = 0.01) but not during congruent trials (β = 1.18^−26^, SE = 6.7^−27^, *t* = 1.78, *p* = 0.07). During incongruent trials, taVNS caused higher power (mean = 0.483 pT^2^/cm, SD = 0.3) compared with sham (mean = 0.467 pT^2^/cm, SD = 0.28). The corresponding model with congruency nested in stimulation revealed no significant effects (all *p* values > 0.14). Furthermore, no effects of stimulation intensity were observed in either of the two clusters (both *p* values > 0.07).

Power at OM gradiometers during the EST was also systematically modulated by congruency, stimulation, and stimulation intensity. The respective CBPT indicated an effect of the predictor stimulation intensity corresponding to a cluster in our data between 2 and 10.5 Hz (*p_cluster_* = 0.0001). The average beta estimate from that cluster was −9.43^−26^ indicating a reduction in power in that frequency range with increasing stimulation intensity. The CBPT also indicated an effect of congruency corresponding to three clusters in our data (Fig. 4*E*,*F*). The first between 2 and 4.5 Hz (*p_cluster_* = 0.0001), the second between 8 and 9 Hz (*p_cluster_* = 0.0085), and the third between 11 and 12.5 Hz (*p_cluster_* = 0.0001). In the first two clusters (between 2 and 4.5 Hz and 8 and 9 Hz) power was higher during incongruent trials (cluster 1, mean = 0.771 pT^2^/cm, SD = 0.57; cluster 2, mean = 0.573 pT^2^/cm, SD = 0.44) compared with congruent trials (cluster 1, mean = 0.767 pT^2^/cm, SD = 0.55, *d_avg_* = 0.18, *d_max_* = 0.18 at 4.5 Hz; cluster 2, mean = 0.57 pT^2^/cm, SD = 0.43, *d_avg_* = 0.24, *d_max_* = 0.27 at 8.5 Hz). In the third cluster between 11 and 12.5 Hz power was higher during congruent trials (mean = 0.513 pT^2^/cm, SD = 0.39) compared with incongruent trials (mean = 0.51 pT^2^/cm, SD = 0.38, *d_avg_* = −0.11, *d_max_* = −0.13 at 12 Hz). The CBPT further indicated a significant effect of stimulation corresponding to a cluster between 2 and 14 Hz (*p_cluster_* = 0.0001; Fig. 4*G*,*H*). Average OM power in that frequency range was lower during taVNS (mean = 0.61 pT^2^/cm, SD = 0.47) compared with sham stimulation (mean = 0.64 pT^2^/cm, SD = 0.55, *d_avg_* = 0.3, *d_max_* = 0.48 at 10.5 Hz). Finally, the CBPT indicated an effect of the congruency-by-stimulation interaction corresponding to two clusters in our data, one between 2 and 4.5 Hz (*p_cluster_* = 0.0001) and one between 10.5 and 12.5 Hz (*p_cluster_* = 0.0001). Nested models were again used to resolve the interaction with each predictor nested within the other and stimulation intensity as fixed effects. Random intercepts across subjects were set as random effects. In the first cluster neither the model with stimulation nested in congruency nor the model with congruency nested in stimulation revealed a significant effect (all *p* values > 0.46). In the second cluster the model with simulation nested in congruency revealed a significant effect of stimulation only during congruent trials (β = −3.42^−26^, SE = 1.4^−26^, *t* = −2.45, *p* = 0.01) but not during incongruent trials (β = −2.5^−26^, SE = 1.4^−26^, *t* = −1.8, *p* = 0.07). Power was lower during taVNS (mean = 0.54 pT^2^/cm, SD = 0.37) compared with sham stimulation (mean = 0.57 pT^2^/cm, SD = 0.47).

**Figure 5.**
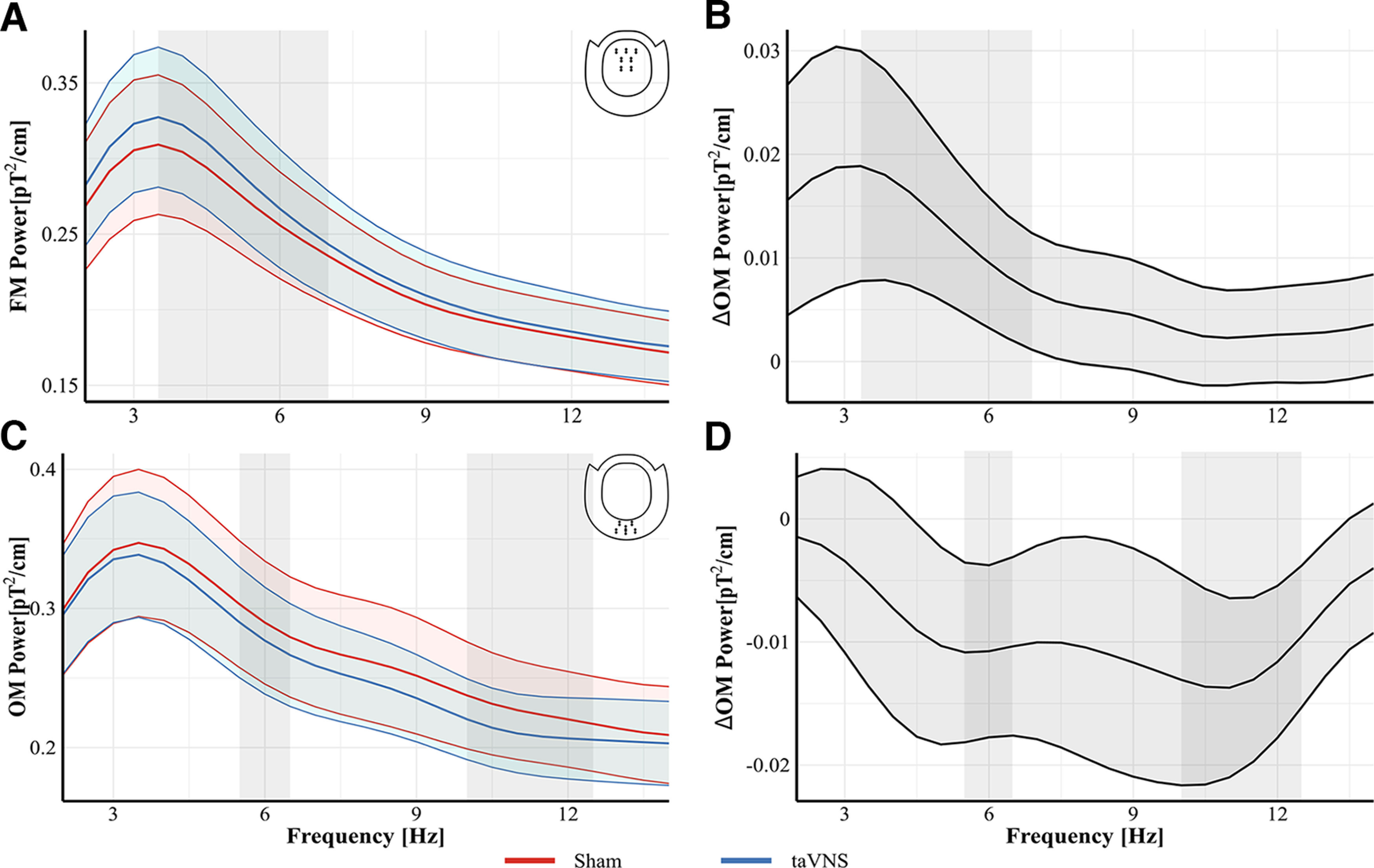
Fourier spectra for conditions (left) and difference waves (right) during the PLRT. ***A***, ***B***, Effect of stimulation at FM sensors during the PLRT. Inset, top right (***A***) Grand average FFT at FM sensors from the PLRT for taVNS (blue) and sham (red). Difference between taVNS and sham trials during the PLRT (***B***). Permutation testing indicated a significant difference between taVNS and sham corresponding to a cluster between 3.5 and 7 Hz (gray rectangle) with higher power during taVNS. ***C***, ***D***, Effect of stimulation at OM sensors during the PLRT. Inset, top right (***C***), Grand average FFT at OM sensors for taVNS (blue) and sham (red). Difference between taVNS and sham trials during the PLRT (***D***). Permutation testing indicated a difference between taVNS and sham corresponding to two clusters between 5.5 and 6.5 and between 10.5 and 12 Hz (gray shaded rectangle) with lower power during taVNS in both clusters. Shaded areas around curves indicate the SEM.

Figure 5 shows the power at FM and OM gradiometers during the PLRT separately for the different stimulation conditions and their respective difference. Power at FM gradiometers during the PLRT was systematically modulated by stimulation. The respective CBPT indicated a significant effect of the predictor stimulation, corresponding to a cluster in our data between 3.5 and 7 Hz (*p_cluster_* = 0.0001; Fig. 5*A*,*B*). Power was higher during taVNS (mean = 0.29 pT^2^/cm, SD = 0.2) compared with sham stimulation (mean = 0.275 pT^2^/cm, SD = 0.19, *d_avg_* = −0.37, *d_max_* = −0.38 at 5 Hz). No effect of stimulation intensity was observed. Power at OM gradiometers during the PLRT was modulated by stimulation and stimulation intensity. The respective CBPT indicated an effect of the predictor stimulation intensity corresponding to a cluster between 2 and 14 Hz (*p_cluster_* = 0.0001). The average beta estimate from that cluster was −3.26^−26^ indicating a reduction in power in that frequency range with increasing stimulation intensity. The CBPT further indicated an effect of the predictor stimulation corresponding to two clusters in our data, one between 5.5 and 6.5 Hz and one between 10 and 12.5 Hz (Fig. 5*C*,*D*). Power in both clusters was lower during taVNS (cluster 1, mean = 0.278 pT^2^/cm, SD = 0.18; cluster 2, mean = 0.211 pT^2^/cm, SD = 0.13) compared with sham stimulation. (cluster 1, mean = 0.291 pT^2^/cm, SD = 0.21, *d_avg_* = 0.34, *d_max_* = 0.34 at 6 Hz; cluster 2, mean = 0.226 pT^2^/cm, SD = 0.17, *d_avg_* = 0.38, *d_max_* = 0.41 at 11.5 Hz).

**Figure 6.**
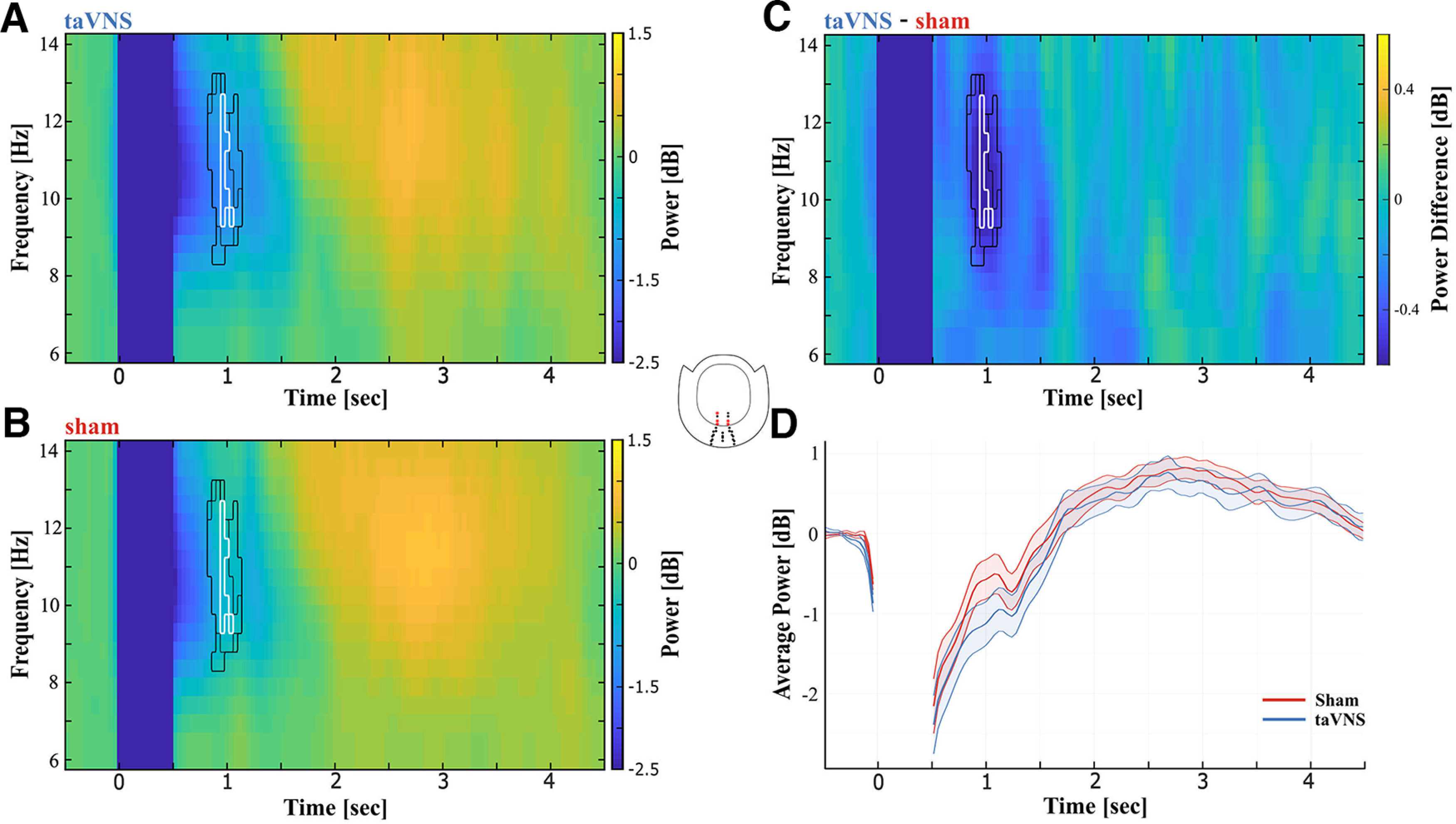
Differences in alpha power at occipital midline gradiometers between taVNS and sham. CBPT was performed for OM gradiometers between 600 and 5000 ms poststimulus. Inset, middle, The analysis indicated a difference between taVNS and sham in the alpha range ∼1000 ms poststimulus at parieto-occipital sensors. Power in this cluster during taVNS was lower compared with sham stimulation. ***A***, TFR from these sensors for taVNS. ***B***, TFR from these sensors for sham. ***C***, Difference between taVNS and sham. Black outlines in ***A–C*** represent the significant time-frequency cluster for each individual sensor. White outlines in ***A–C*** represent the overlap of time-frequency clusters that were significant in all five sensors. ***D***, Time course of averaged alpha power between 8.5 and 13 Hz. The period between 0 and 500 ms was not considered in the analysis and was blanked out in the figure accordingly. The shaded area around curves indicates SEM.

Power at OM Gradiometers during the EST was also modulated by taVNS after stimulation offset. The CBPT between 600 and 5000 ms poststimulus indicated a difference between taVNS (Fig. 6*A*) and sham (Fig. 6*B*) corresponding to a cluster in the alpha range between ∼8.5 and 13 Hz, ∼1000 ms after stimulus onset and 500 ms after stimulation offset at parieto-occipital sensors (*p_cluster_* = 0.034). During taVNS, alpha power was lower compared with sham (Fig. 6*C*). Figure 6*D* shows the time course of the averaged alpha power between 8.5 and 13 Hz. Power values from the time period between 0 and 500 ms were cut from the data for visualization to indicate that this period was not included in this analysis. The CBPT on TFR data during the PLRT indicated no significant difference.

### Relationship between parameters across tasks

Finally, we examined whether electrophysiological and pupillometric measures in one task could be predicted by the corresponding measure in the other task. For the pupil differences during the EST, CBPT indicated that neither Δpupil.plrt1 nor Δpupil.plrt2 were significant predictors for Δ*PD_EST_*. For the pupil differences during the PLRT, CBPT indicated also no significant effect of Δpupil.est.

For the FM power differences during the EST, CBPT indicated that Δfm.plrt was a significant predictor over the entire analyzed frequency range between 2 and 14 Hz (*p_cluster_* = 0.0001). The average positive beta estimate from within that cluster of 1.37^−26^ indicated that subjects with higher FM power differences during the PLRT (i.e., more power during taVNS) also exhibited higher FM power differences during the EST. Vice versa, for FM power differences during the PLRT the CBPT indicated an effect of Δfm.est corresponding to a cluster between 2 and 14 Hz in our data (*p_cluster_* = 0.0001). Again, the positive average beta estimate of 4.7^−27^ indicated that subjects with higher-power differences in the EST also exhibited higher-power differences in the PLRT. For the OM power differences during the EST, the respective CBPT indicated an effect of Δom.plrt1 corresponding to a cluster between 2 and 14 Hz (*p_cluster_* = 0.0001) with an average beta estimate of −1.12^−26^. This negative beta indicates that subjects who showed lower OM power during the PLRT ∼6 Hz also exhibited lower OM power during the EST. Apart from that, the CBPT indicated no effect of Δom.plrt2. Finally, the CBPT on OM power differences during the PLR indicated an effect of Δom.est corresponding to a cluster between 2 and 6.5 Hz (*p_cluster_* = 0.0002). The average beta estimate was −6.2^−27^, indicating again that subjects with lower OM power in the EST also exhibited lower OM power in the PLRT.

## Discussion

In this study, we show for the first time that phasic event-related taVNS systematically modulates behavioral, pupillary, and electrophysiological parameters of LC-NA activity during cognitive processing. We show that taVNS (1) increased pupil dilation and improved performance in an EST, (2) reduced the amplitude and delayed the onset of the pupil constriction of the PLR task, and (3) increased task-related theta and alpha power while reducing occipital alpha power. These results extend previous work on taVNS that has solely used task-free settings (Sharon et al., 2021; Urbin et al., 2021; D'Agostini et al., 2023) and demonstrate for the first time that taVNS systematically modulates the pupil light reflex. Finally, we show that electrophysiological parameters were associated across tasks. Higher taVNS-induced FM power in the EST was associated with higher taVNS-induced FM power in the PLRT, and lower OM power in the EST during taVNS was associated with lower OM power in the PLRT during taVNS.

Despite the stimulation duration, our study matched former taVNS studies in terms of stimulation amplitude, pulse width, and frequency. It is thus likely that longer stimulation durations like 30 s (Warren et al., 2019; Burger et al., 2020a) or continuous stimulation (Keute et al., 2019; D'Agostini et al., 2021, 2022) only modulate tonic LC firing without an effect on phasic LC activity.

Behavioral results during the EST show the expected Stroop effect, that is, an increase in RT and a decrease in accuracy during incongruent trials, indicating processing costs for conflict resolution (Botvinick et al., 2001). More importantly, we additionally observed a stimulation-specific effect on accuracy during the EST. During taVNS, subjects responded more accurately compared with sham stimulation. Remarkably, this effect was apparent despite the notable occurrence of numerous subjects achieving or closely approaching ceiling levels, denoting perfect accuracy (i.e., 100%). This might have attenuated the stimulation effect on behavioral performance. Tasks that are more difficult, or subject populations already exhibiting impaired cognitive control, might show stronger benefits on behavioral accuracy from taVNS. Interestingly, this behavioral improvement was accompanied by a stimulation-specific increase in FM theta and alpha power. Reaction times, however, were not affected by taVNS. This might be because of the fact that our sample consisted of relatively young, healthy adults, who already performed at or near optimal levels with little to no room for improvement in reaction time. Again, subject groups with already impaired cognitive control might show improved reaction times by taVNS. However, this is only speculative and needs further investigation.

Our observations further underline the potential of FM theta power as an electrophysiological marker of taVNS during cognitive processing. The increase in FM alpha power, however, was somewhat surprising, as recent studies suggest an overall reduction in cortical alpha power (Lewine et al., 2019; Sharon et al., 2021). In these studies, alpha was viewed as an idling state of low cortical arousal. However, this notion is debated (Cooper et al., 2003), for instance, by studies showing increased alpha power with increasing working memory load (Klimesch, 1999; Jensen et al., 2002). Additionally, internally directed attention has been associated with higher alpha power at frontal and central locations (Cooper et al., 2003). Thus, the observed increase in alpha power following taVNS could be because of direct noradrenergic modulation or reflect a shift toward more internally directed attention as an indirect effect of increased LC-NA activity. However, as we did not ask participants about their focus of attention, this notion warrants further investigation. Finally, we replicated the decrease in occipital alpha power following taVNS (Sharon et al., 2021) but now in an active cognitive task with visual stimulation. We observed these taVNS-specific effects on occipital alpha power during both the EST as well as the PLRT. In both tasks, power in the alpha range at OM gradiometers was reduced following taVNS compared with sham stimulation. Although the passive PLRT might be more comparable to previous work that applied taVNS in a task-free environment (Sharon et al., 2021), we extend previous findings by the observation that a comparable decrease can also be observed during cognitive processing. This provides further evidence for the potential of occipital alpha power as an indicator of taVNS efficacy. However, these results must be interpreted with care because of our correction of the stimulation artifact. For that, we removed contaminated portions of the data and interpolated these gaps using an autoregressive function. As we used monophasic pulses, which seem to cause longer-lasting artifacts compared with biphasic pulses (Keatch et al., 2022), we had to remove larger chunks of the original data. This might have decreased our signal-to-noise ratio, which could explain the small effect sizes observed in the theta range for the congruency effect. Our observations in the alpha range however are robust and replicate recent findings (Sharon et al., 2021). Therefore, we are optimistic that we did not overcompensate during our artifact rejection approach.

Our pupil recordings during the EST show the well-known incongruency effect, that is, stronger pupil dilation during incongruent trials compared with congruent trials (Grueschow et al., 2020, 2021), further supporting the involvement of the LC-NA system in the resolution of response conflicts. Most importantly, however, we also observed a strong stimulation-specific effect on pupil diameter. We observed increased pupil dilation following taVNS, suggesting a stimulation-induced increased level of noradrenergic tone. The LC shows two discernable activity patterns, (1) a tonic baseline activation and (2) a phasic burst-like activation following salient or behaviorally relevant stimuli. Tonic LC activity is related to baseline PD, whereas the phasic activation is reflected in transient increases in PD (Aston-Jones and Cohen, 2005). One could argue that the phasic stimulation used in our study, in contrast to the often used 30 s of taVNS, could better mimic and thus amplify the phasic activation pattern of the LC. This then leads to an amplification of phasic LC activations after relevant stimuli, which then causes an observable, transient increase in pupil diameter. The temporal dynamic of longer stimulation intervals is closer in resemblance to the tonic activation pattern without the increased effect on transient pupil dynamics. The pupil diameter is controlled by the interplay of two antagonistic muscles—the sympathetically innervated pupil dilator muscle and the parasympathetically innervated pupil sphincter muscle. Sympathetic neurons innervating the dilator muscle originate in the superior cervical ganglion. Parasympathetic innervation stems from the ciliary ganglion, which in turn receives input from the EWN (Eckstein et al., 2017; Hall and Chilcott, 2018). NA plays a key role in both systems. It reduces activity in the EWN through inhibitory alpha_2_-adrenoreceptor and activates the pupil dilator muscle via excitatory alpha_1_-adrenoreceptors (Samuels and Szabadi, 2008a, b; Hall and Chilcott, 2018). Thus, either activation of the sympathetic or inhibition of the parasympathetic route can lead to pupil dilation. This leads to three potential explanations for our results. First, through sole central inhibition of the EWN. In this case, the pupil sphincter muscle would receive less input from the EWN during taVNS. The balance between both muscles would then shift towards relatively stronger activity in the sympathetic dilator muscle. Second, through sole excitation of the pupillary dilator muscle. In this case, taVNS would only activate the sympathetic part of the pupil's innervation. However, this is highly unlikely as NA would have to be released directly at the pupil only and not in the brain. The third possible explanation is the combination of both factors, that is, simultaneously increased activity in the sympathetic and increased inhibition of the parasympathetic route. In our eyes, the first explanation is the most plausible. A study in mice investigated the pathways necessary for the pupil dilation following direct phasic LC stimulation. After surgical removal of the superior cervical ganglion and pharmacological blockage of alpha_2_-adrenoreceptors in the EWN through yohimbine, no pupil dilation in response to stimulation was observed. Importantly, ∼1 h after yohimbine application, the stimulation-induced pupil dilation returned (Liu et al., 2017). This supports the idea of increased noradrenergic EWN inhibition as cause for the increased pupil dilation in our study. The same mechanism holds for the observed reduction of the PLR. Following the influx of light, activity in the EWN increases, which leads to a constriction of the pupillary sphincter muscle. During taVNS, the increased NA concentration likely inhibited activity in the EWN, which, in turn, reduced pupil constriction. The observed increase in latency of the PLR is also in line with a pharmacological study, showing that NA reuptake inhibitors prolonged the latency of PLR (Theofilopoulos et al., 1995).

Although our pupillometric results are consistent with an increased LC activation, it is necessary to note that the LC-NA system is possibly not the only neuromodulatory system involved. The NTS is an important relay station in the brainstem, with widespread connections to, for example, the raphe nuclei or limbic and forebrain areas (Krahl and Clark, 2012; Frangos et al., 2015). Especially, cholinergic projections from the basal forebrain are also known to mediate pupil dilation in rodents (Reimer et al., 2016; Mridha et al., 2021). Hence, a cholinergic interaction cannot completely be ruled out. Although these transmitter systems have most likely only secondary effects, their potential influence warrants future investigation.

Another limitation may concern the stimulation itself. Electrophysiological recordings during active stimulation suffer from the presence of stimulation artifacts several orders of magnitudes stronger than the actual brain activity, and MEG is especially susceptible to these artifacts. Thus, the number of studies that apply stimulation while simultaneously recording brain activity is limited, and only a handful of MEG studies that applied taVNS during critical periods (e.g., onset of target stimuli) have been conducted so far (Lehtimäki et al., 2013; Hyvärinen et al., 2015; Keatch et al., 2022). Different artifact correction mechanisms, like temporal signal space separation (Taulu and Simola, 2006), have been applied, but nonlinear components of the artifact make it hard to capture. Although previous studies on simultaneous MEG and taVNS used temporal signal space separation as an artifact cleaning method (Lehtimäki et al., 2013; Hyvärinen et al., 2015), we had to realize that this method was not adequately applicable for our data as it was unable to sufficiently remove the artifact from our MEG recordings. Accordingly, we implemented an artifact-cleaning approach as demonstrated in Keatch et al. (2022), which involved the exclusion and subsequent interpolation of contaminated time points. This method has been shown to retain a good amount of information in the signal, especially at lower frequencies. However, as interpolation bears the risk of altering the original data, this method has to be used with caution. Hence, better methods are required to sufficiently clean the electrical artifact to better estimate online taVNS effects. Future work is also needed to determine, whether phasic taVNS modulates other indirect LC-NA markers, for instance salivary alpha-amylase (Giraudier et al., 2022).

In conclusion, we show compelling evidence that phasic event-related taVNS modulates pupil dilation as well as theta and alpha power likely through central noradrenergic modulation. In contrast to previous work using task-free taVNS paradigms (Sharon et al., 2021; Urbin et al., 2021; D'Agostini et al., 2023), we show that these metrics can be obtained while being engaged in a cognitive task. This has the added bonus of investigating potential taVNS effects on behavioral performance as we have done here during a cognitive control task. Finally, we also show that the PLR can effectively be used to index taVNS effects, providing an additional, easy-to-use biomarker for future taVNS studies.
